# Electrophysiological Mechanism of Attention of Sleep Deprivation: Evidence From Event-Related Potentials (ERP) Data

**DOI:** 10.7759/cureus.33464

**Published:** 2023-01-06

**Authors:** Shengjun Wu, Peng Yue, Lin Wu, Chaoxian Wang, Xinxin Lin, Xinhong Li

**Affiliations:** 1 Military Medical Psychology, Air Force Medical University, Xi'an, CHN; 2 Unit 94955 of PLA, Nantong, CHN; 3 General Practice, Tangdu Hospital, Air Force Medical University, Xi'an, CHN

**Keywords:** cognitive function, event-related potentials, attention, electroencephalogram, sleep deprivation

## Abstract

Introduction: The purpose of this study is to investigate the effects of sleep deprivation on individual attentional function and related electrophysiological mechanisms.

Methods: Twenty healthy men who were deprived of sleep for 24 h were evaluated by selective attention test, persistent attention test, and event-related potentials (ERP) experiment.

Results: After 24 h of sleep deprivation, the subjects' selective attention decreased, mainly manifested as prolonged response time, decreased motion stability, increased rate of neglect error, decreased sustained attention, prolonged latency of P300 at Cz (*p*=0.001), and decreased amplitude (*p*=0.000).

Conclusion: After 24 h of sleep deprivation, the attentional ability decreased significantly, and behavioral and ERP indicators showed certain changes.

## Introduction

In recent decades, with the change of social conditions, the increasing pace of society and the increasing pressure of work have led to a growing problem of sleep deprivation, which has become an important social and health problem, and people have begun to pay more attention to this area. Therefore, the problem of sleep deprivation has become increasingly prominent, prompting people to pay more attention to this area. Studies have shown that sleep is related with emotion [1-2]. Many studies also verified that loss of sleep has adverse effects on personal life, work performance, and medical accidents to varying degrees [3-5]. Sleep deprivation can lead to an increase in the incidence and potential incidence of motorcycle accidents [6].

Sleep deprivation can affect all aspects of cognitive function, such as motivation impairment, irritability, difficulty in concentrating, reduced or lost alertness, memory decline, slow action, slow reaction, increased errors in judgment, and even audio-visual hallucinations. Due to the "focusing" effect of attention, the influence on attention may indirectly affect other cognitive functions. Smith's study of 24-h sleep deprivation found that participants' scores on visual tracking, auditory alertness, and repeated number alertness were significantly decreased, while their scores on logic tests and time estimation tests requiring reasoning were not significantly changed. It is suggested that 24-h sleep deprivation mainly damages tasks requiring high concentration and quick reaction [7]. Patrick et al. made similar findings [8]. It is found that after 36 h of sleep deprivation, accuracy decreased by 2.23% and reaction time increased by 8.82% which indicated that the cognitive function was significantly affected [9]. Pasula et al. found that younger adults showed a significantly larger drop compared with older adults in verbal encoding and in visuospatial displacement following 36 h sleep deprivation [10]. However, Honn et al. found that after sleep deprivation, the 20% slowest response times (RTs) were significantly increased but the semantic encoding remained at baseline level [11]. Nakano et al. examined changes in posture, alertness, and anal temperature during 19 h of sleep deprivation. Alertness was measured by the ratio of alpha wave activity intensity between closed eyes and open eyes, and posture swing by the shift of the center of gravity of both feet. When eyes were closed, the postural swing increased the next morning, and six of the eight subjects also had the maximum postural swing during the three-hour period when their anal temperature was the lowest [12].

Partial sleep deprivation may also affect attention. Mishra et al. found that partial sleep deprivation had the similar impact on cognition and psychomotor vigilance [13]. Peszka and Harsh explored the effect of sleep deprivation on event-related potentials (ERP) in non-rapid eye movement sleep (NREM) period, and found that the ERP amplitude in NREM period increased with the increase of sound stimulation intensity and sleepiness degree. Sleep deprivation was more likely to lead to rapid fall asleep, more difficult to wake up and larger spindling waves. The amplitudes of N350, N550, and P900 were higher, especially when the sound intensity was higher. The amplitude of P220 has not changed. These results are consistent with the view that ERP in NREM period reflects arousal level [14]. Drummond et al. explored the fMRI data of 13 subjects before and after 35 h sleep deprivation by a new paradigm, and the results indicated that their work performance was moderately impaired. Moreover, the activity of prefrontal and parietal lobes, especially the right side, increased after sleep deprivation, and the activities of the left inferior frontal lobe were correlated with the subjective sleepiness after sleep deprivation. Parietal lobe activity was associated with difficulty of initial memory task after sleep deprivation, and increased activity in many brain regions was associated with attentional control after sleep deprivation. These results suggest that such distributive tasks may require more attentional resources after sleep deprivation, such as operational tracking and attentional retention [15].

The negative effects of sleep deprivation on attention ability have been revealed by many studies, but there is a lack of in-depth studies, such as the effects on selective attention and persistent attention, and whether the self-assessment results of subjects are parallel. What stages of selective attention are affected? Are there consistent effects on attentional tasks of different difficulty levels? ERP studies are on the rise, and ERPs such as P300 are reliable indicators to evaluate attention ability, but they are seldom used in sleep deprivation studies. Combined with the ERP technology of visual and auditory selective attention, the dynamic process of sleep deprivation affecting selective and sustained attention is investigated. Therefore, this study focused on the attention ability, combined with the selective attention package test, persistent attention test, visual and auditory selective attention ERP test, to explore the characteristics of the change of attention ability of healthy young subjects during 32 h of sleep deprivation.

## Materials and methods

Participants and design

Participants

From July to August 2014, 20 undergraduate students aged 18-22 years (mean age = 21.15 years, standard deviation - SD = 1.78 years; 10 males and 10 females) from a military medical university were recruited to participate in this study. They were all right-handed, with normal vision and hearing, without color blindness or color weakness, and without any history of mental or neurological diseases. The results of 19 participants were analyzed at the end of the study because one volunteer dropped during the study due to conflicts between the experimental process and their course schedules. This study was approved by the Ethics Committee of Tangdu Hospital (2014-03-03) and had been registered at ClinicalTrials.gov (NCT02420470, http://www.clinicaltrials.gov/). All procedures were conducted according to the principles set forth in the Declaration of Helsinki. All participants gave informed consent and were paid four weeks after the experiment.

Design

The cognitive tests were completed at 8 am on the experimental day. The subjects did not sleep at that night and the next day. The measurements were repeated at 8 am on the next day, and the sleep deprivation ended at 4 pm. The attention test and ERP experiment were performed before and after the experiment.

Cognitive task

Selective Attention Test

The subjects were asked to make different keystroke responses according to different stimuli successively appearing on the screen. According to the characteristics of stimulus (shadowed or unshadowed), the interval of stimulus (fixed or unfixed), and the response mode (symmetrical or asymmetric response), the three groups were divided into six sub-tests. The number of operational errors, the number of neglectable errors, and the error rate were obtained by recording the subjects' buttons and reactions each time.

There are six modes of selective attention task, and the test principles are corresponding in pairs, namely A1, A2, B1, B2, C1, and C2 modes. During the test, the subjects sit in front of the monitor with an overlooking angle of about 10-15 degrees, and place both hands on the keyboard gently without lifting the fingertips from the key surface when pressing the key.

In mode A, subjects performed four different keystroke responses according to four different stimuli, with stimulus materials as "1," "2," "3," and "4." The stimulus presentation time was 100 ms, the effective timing area was 2000 ms, and the stimulus interval was 4000 ms, presenting 60 times in total. In A1 mode, the size of the stimulus material is 40 × 80 pixels. The difference between A2 and A1 is that the stimulus pixels are evenly spaced at a ratio of 1:3 in the vertical and horizontal directions. In mode B, subjects made four different button responses according to the two stimuli appearing in the left or right visual field, and the stimulus materials were "2" and "3." The difference between B1 and B2 was the different button rules. The button rule in B1 mode is "2-left finger press D, 3-left index finger press F, 3-right index finger press J, and 2-right middle finger press K." In B2 mode, "2-left finger press D, 3-left index finger press F, and 2-right index finger press J." The stimulus size, times of presentation and time constant were set in the same mode as A. In mode C, subjects responded to two different keys according to two different stimuli with stimulus materials of "2" and "3." In C1 mode, the interval time of stimulus presentation was 4000 ms, while in C2 mode, the interval time of stimulus presentation was randomly distributed in the range of 3000-5000 ms. Other constants are set in the same mode as A.

Sustained Attention Test

The subjects were asked to place the cursor in the middle of a constantly moving ball on the screen, and the program continued to record the distance between the two (in pixels, with the 17-inch full-screen mode set to 800 * 600 pixels) for 30 min.

ERP Test

Auditory Go-Nogo paradigm (target stimulus: non-target stimulus = 1:1) was used. Presented by STIM stimulus render. The sound stimulus is pure tone of 1000 and 2000 Hz, with sound intensity of 20 dB, duration of 500 ms, and inter-stimulus interval of 1000 ms. When the high-frequency stimulus occurs, press the "3" key on the response key box.

Instruments

**Figure 1:**
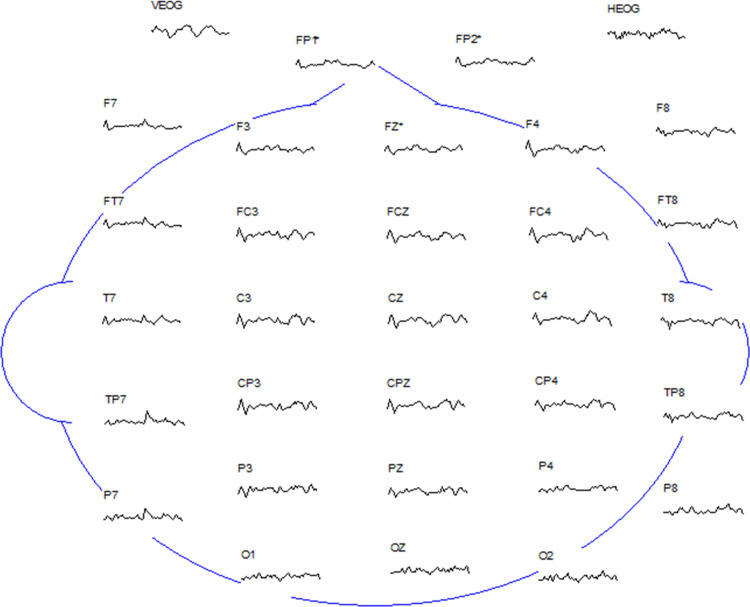
International standard 10-20 system electrode placement method.

The electroencephalogram (EEG) was recorded by ESI-32 recording system. The international standard 10-20 system electrode placement method is adopted to place the electrodes, as shown in Figure 1. Vertical and horizontal eye movements were recorded at the same time, and reference electrodes were placed at the mastoid process behind the left and right ears. The EEG gain was 1000 times, sampling frequency was 100 Hz, band pass was 0.1-40 Hz, and resistance between electrode and scalp was 0-25 kΩ.

Reaction rules

Power spectrum estimation method can be divided into classical spectrum estimation method and modern spectrum estimation method. Compared with classical spectral estimation methods such as fast Fourier Transform algorithm (FFT), modern spectral estimation method has the advantages of smooth spectral curve and high resolution. It can be divided into parameter model spectral estimation and non-parameter model spectral estimation. The autoregressive (AR) model is one of the parameter model spectral estimation. AR was widely used in EEG experiments. According to this model, the current output is the weighted sum of the current input and the past output, which can be expressed by Formula 1:

$x(n)=\sum_{r=1}^{p}{-a_rx(n-k)+u(n)}$…………………… Formula 1

Where x is the signal matrix, a is the parameter, u is the noise series, and p is the order of the model. Burg algorithm can be used to solve the equation to obtain model parameters, and then Formula 2 can be used to obtain the spectrum:

$P_X(k)=\frac{\sigma^2}{\left|1+\sum_{r=1}^{p}{a_rW_N^{-kr}}\right|^2}$……………………… Formula 2

where σ2 is the variance of noise series. In this study, a nine-order AR model was used to analyze EEG signals in the last 3 s. The extracted power spectrum amplitude of sensorimotor rhythm corresponds to the length of the red rectangle bar on the screen as visual feedback signal. Subjects adjust their mental activities according to the length of the rectangle bar to achieve the purpose of controlling the power spectrum amplitude of sensorimotor rhythm.

Statistical analysis

Data were entered using EpiData 3.1, which is a statistics software and its manufacturer is Odense, Denmark which is a non-profit organization, and the research team includes Jens M. Lauritsen, Mark Myatt, and Michael Bruus and statistical analysis was performed on SPSS 23.0 (IBM Corp., Armonk, NY). Descriptive analysis, paired t test, Anova and Manova1, Kolmogorov-Smirnov normal distribution test, and Bartlett variance homogeneity test were performed on the data obtained; p<0.05 indicated statistical difference.

## Results

Comparison of selective attention before and after sleep deprivation

The performance of selective attention test has five indicators including reaction time (RT), action stability (AS), omission error ratio (OER), commission error ratio (CER), and errors ratio (ER). RT refers to the time for the subject to make the correct button response. In this ERP experiment, the subjects need to make button response according to the cognitive task. Pressing the button in advance, pressing the wrong button, or not pressing the button after the timing area are all wrong responses, and AS is the mean of SD of reaction time in the case of correct response. OER refers to the rate of the participants' omission error, which refers to the subjects' failure to respond to the stimulus presented on the screen within a specified period of time. CER refers to the rate of the subjects' key errors. In this ERP experiment, if the subject presses the button in advance, presses the wrong key or fails to press the button after the timing area, etc., all of them belong to the wrong response. ER is the wrong response rate, which refers to the percentage of the wrong response in the total number of keys. Error rate refers to the sum of neglect error rate and operational error rate.

The paired t-test was used to test the performance of five operations in six measurement modes before and after sleep deprivation. The results are shown in Table 1. The results showed that AS in A1 mode, AS and OER in B1 mode, OER in B2 mode, TR, AS and OER in C1 mode, and RT and OER in C2 mode were significantly different before and after sleep deprivation, suggesting that after sleep deprivation, the response time of subjects was significantly prolonged, the stability of action decreased, and the rate of neglect error increased significantly.

**Table 1:** Operational performance of six selective attention tasks pre- and post-sleep deprivation (n=19, M±SD). RT, reaction time; AS, action stability; OER, omission error ratio; CER, operational error ratio; ER, errors ratio

Mode	Index	Stage		t	p
		Pre	Post		
A1	RT	0.499±0.063	0.521±0.079	-1.822	0.072
	AS	0.142±0.055	0.173±0.063	-3.101	0.002
	ER	0.058±0.055	0.071±0.051	-1.450	0.149
	OER	0.011±0.042	0.020±0.030	-1.459	0.147
	CER	0.047±0.039	0.051±0.049	-0.534	0.594
A2	RT	0.530±0.066	0.538±0.072	-0.682	0.494
	AS	0.152±0.067	0.175±0.081	-1.831	0.069
	ER	0.055±0.052	0.070±0.054	-1.674	0.096
	OER	0.011±0.046	0.020±0.038	-1.262	0.209
	CER	0.044±0.034	0.050±0.040	-0.956	0.341
B1	RT	0.527±0.082	0.532±0.093	-0.337	0.736
	AS	0.147±0.039	0.167±0.043	-2.882	0.005
	ER	0.053±0.046	0.064±0.047	-1.399	0.164
	OER	0.002±0.010	0.008±0.010	-3.550	0.001
	CER	0.053±0.046	0.056±0.044	-0.394	0.694
B2	RT	0.508±0.065	0.526±0.074	-1.529	0.129
	AS	0.149±0.031	0.159±0.059	-1.255	0.177
	ER	0.062±0.042	0.069±0.047	-0.929	0.354
	OER	0.001±0.004	0.011±0.024	-3.439	0.001
	CER	0.061±0.042	0.058±0.042	0.423	0.673
C1	RT	0.389±0.057	0.414±0.087	-2.011	0.020
	AS	0.118±0.057	0.145±0.063	-2.659	0.046
	ER	0.081±0.120	0.101±0.148	-0.878	0.381
	OER	0.002±0.005	0.015±0.024	-4.437	0.000
	CER	0.080±0.118	0.086±0.151	-0.262	0.794
C2	RT	0.380±0.065	0.410±0.083	-2.381	0.019
	AS	0.122±0.053	0.139±0.048	-1.989	0.049
	ER	0.103±0.145	0.113±0.138	-0.418	0.677
	OER	0.003±0.005	0.015±0.017	-5.666	0.000
	CER	0.101±0.146	0.088±0.140	0.538	0.592

Comparison of persistent attention before and after sleep deprivation

**Figure 2:**
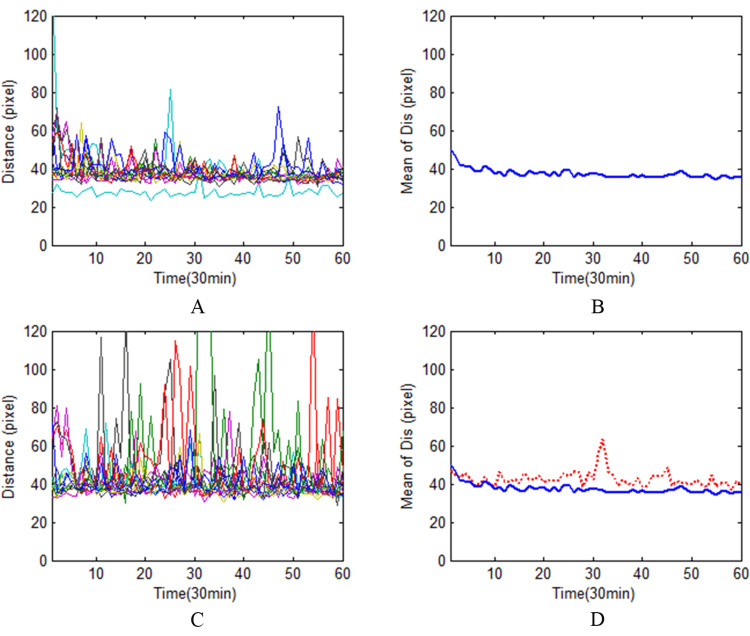
Comparison of distance between cursor and ball pre- and post-sleep deprivation. A is the change of distance between the cursor and the ball of each subjects over time from 0 to 30 min before sleep deprivation. B is the mean of distance between the cursor and the ball of each subjects over time from 0 to 30 min before sleep deprivation. C is the change of distance between the cursor and the ball of each subjects over time from 0 to 30 min after sleep deprivation. D is the mean of distance between the cursor and the ball of each subjects over time from 0 to 30 min after sleep deprivation.

The distance between the cursor and the ball reflects the tracking speed, and its variance reflects the stability of the motion. The curve of distance over time directly reflects the change of sustained attention level. According to the operation curves and their average curves, it can be seen that the average curve after sleep deprivation is significantly higher (Figure 2C,D) than that before sleep deprivation (Figure 2A,B). Paired t test showed significant difference in distance before and after sleep deprivation (p=0.018), but insignificant difference in stability (p=0.176), as shown in Table 2.

**Table 2:** Comparison of the distance and stability between the cursor and the ball pre- and post-sleep deprivation (n=19).

index	Stage of sleep deprivation		t	p
	Pre	Post		
Distance	40.867±5.884	44.902±7.887	-2.426	0.018
Stability	35.996±17.523	42.044±19.445	-1.367	0.176

**Figure 3:**
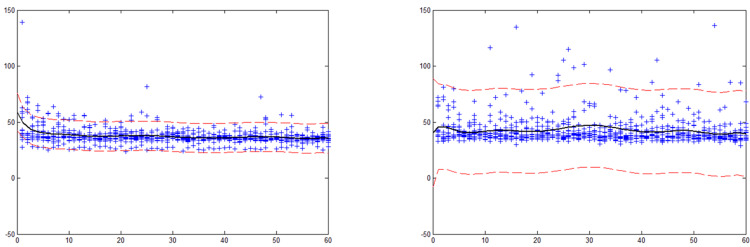
Characteristics of the distance between the cursor and the ball pre- (left) and post- (right) sleep deprivation with test duration. The horizontal axis is the test duration, 30 min in total. The area inside the dotted line has a 95% confidence interval.

The distance between the cursor and the ball reflects the tracking speed, and its variance reflects the stability of the motion. The curve of distance over time directly reflects the change of sustained attention level. In order to investigate the change of distance before and after sleep deprivation, 10 order curve fitting was done. The results showed that the distance fluctuation after sleep deprivation is more than before sleep deprivation, and its 95% confidence interval is larger than the other (Figure 3 and Table 3).

**Table 3:** Ten-order curve fitting coefficient of distance between cursor and ball before and after sleep deprivation (n=19). X^n^, fitting the coefficient of the NTH term in the polynomial. Distance_1_, distance between the cursor and the ball before sleep deprivation. Distance_2_, distance between the cursor and the ball after sleep deprivation

Order	Distance_1_	Distance_2_
X^10^	3.8342e-014	-1.2652e-012
X^9^	-1.9542e-011	3.8628e-010
X^8^	3.7676e-009	-5.0167e-008
X^7^	-3.8022e-007	3.6118e-006
X^6^	2.2591e-005	-0.00015748
X^5^	-0.00082725	0.0042678
X^4^	0.018826	-0.070999
X^3^	-0.26152	0.68905
X^2^	2.1301	-3.4711
X^1^	-9.5558	6.6963

Comparison of ERP after sleep deprivation

**Figure 4:**
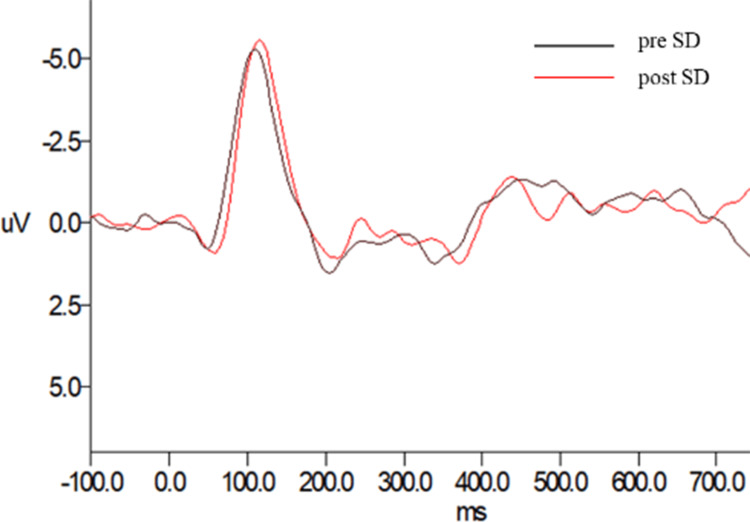
Comparison of total mean waveform of auditory P300 pre- and post-sleep deprivation.

The ERP waveforms of each subject in various modes (band pass: 1-30 Hz, time domain: 100-800 ms) were obtained after the original EEG data were removed from artifact, filtered, baseline correction, and stacked averaging. The total average ERP waveforms of all subjects before and after sleep deprivation in various modes were obtained after stacked averaging again, which were all taken from THE Cz electrode. The results showed that the latency of the total mean waveform of P300 after sleep deprivation was longer than that before sleep deprivation, and the amplitude was lower than that before sleep deprivation (Figure 4).

Paired t-test was used to extract latency and amplitude of the total mean waveforms of all recording electrodes before and after sleep deprivation. The results of comparison showed that after sleep deprivation, the latency of P300 was significantly prolonged (61.167 ± 81.840, p=0.001), and the amplitude of P300 was significantly decreased (-0.208 ± 0.193, p=0.000).

## Discussion

There are many researches on the influence of sleep deprivation on EEG signal, which show that the degree of influence and the influencing factors of EEG signal in different wave bands under sleep deprivation have their own characteristics.

Previous studies focused on the effects of sleep deprivation on resting state EEG. Makeig et al. investigated the behavior and EEG performance of subjects in 42-h sleep deprivation when they completed the tracking task, and found that subjects had an 18-second standstill period with no response, during which the EEG amplitude of the parietal region was both high at 4 and 40 Hz. The experiment confirmed that the awake control in the behavioral sense was not only related to the emergence of spindle wave at 12-14 Hz. It is also associated with θ waves [16]. Many studies found that increased α and θ energy values reflected the subjective sleepiness [17-18]. Zhang et al. found power in the β band (13-30 Hz) increased, whereas power in the θ (4-8 Hz) and α (8-13 Hz) band significantly decreased after sleep deprivation [19].

Sleep deprivation also has an effect on attention. A meta study summarized the performance decrements in sustained attention during sleep deprivation [20]. Zhang et al. found that EEG signal is a sensitive and effective indicator to evaluate sleep in brain functional organization [21]. Liu et al. found the astronauts showed smaller amplitude of the P300 under the sleep deprivation condition [22]. In a 36 h sleep deprivation, it s found that the P300 amplitudes decreased, and the cognitive impairment during 38 h of sleep deprivation was mainly in terms of vigilance and reaction time [23]. Kusztor et al. found that 24 h of sleep deprivation resulted in declined sustained attention and reduced P300 amplitudes [24]. In the study of 30-36 h of sleep deprivation, Díaz-Leines et al. found the delayed latencies of P300 and MMN wave in the neurophysiological tests, and the significant correlations with positive direction between P300 latency and words in noise and music discrimination. All these studies indicated the correlations of change of P300 and sleep deprivation.

## Conclusions

In the current study, it was found that after 24 h of sleep deprivation, subjects showed behavioral characteristics such as prolonged reaction time, decreased motor stability, and increased error rate. Meanwhile, ERP results showed decreased P300 amplitude and prolonged latency, indicating a decreased level of selective attention processing. The results were consistent with the attention energy model of Kahneman et al. The limitation of this study is that the sample size of subjects is not large enough, which may affect the reliability of the results to some extent. In future studies, it is necessary to continue to expand the sample size to seek more reliable results.
